# Sensitivity of Heterogeneous Marine Benthic Habitats to Subtle Stressors

**DOI:** 10.1371/journal.pone.0081646

**Published:** 2013-11-28

**Authors:** Iván F. Rodil, Andrew M. Lohrer, Simon F. Thrush

**Affiliations:** 1 Departamento de Ecología y Biología Animal, Universidad de Vigo, Vigo, Spain; 2 National Institute of Water and Atmospheric Research, Hamilton, New Zealand; University of Gothenburg, Sweden

## Abstract

It is important to understand the consequences of low level disturbances on the functioning of ecological communities because of the pervasiveness and frequency of this type of environmental change. In this study we investigated the response of a heterogeneous, subtidal, soft-sediment habitat to small experimental additions of organic matter and calcium carbonate to examine the sensitivity of benthic ecosystem functioning to changes in sediment characteristics that relate to the environmental threats of coastal eutrophication and ocean acidification. Our results documented significant changes between key biogeochemical and sedimentary variables such as gross primary production, ammonium uptake and dissolved reactive phosphorus flux following treatment additions. Moreover, the application of treatments affected relationships between macrofauna communities, sediment characteristics (e.g., chlorophyll *a* content) and biogeochemical processes (oxygen and nutrient fluxes). In this experiment organic matter and calcium carbonate showed persistent opposing effects on sedimentary processes, and we demonstrated that highly heterogeneous sediment habitats can be surprisingly sensitive to subtle perturbations. Our results have important biological implications in a world with relentless anthropogenic inputs of atmospheric CO_2_ and nutrients in coastal waters.

## Introduction

Major disturbance events are likely to affect the structure and function of ecological communities with large and persistent consequences [1]–[4]. However, recent studies have demonstrated that subtle sublethal stressors can also influence ecosystem functioning [5]–[7] and that small changes matter [8]. Thus, for example, poleward shifts in species distributions have been linked to small gradual increases in annually averaged temperatures globally, despite the comparatively greater frequency and magnitude of temperature fluctuations on short time scales [9], [10].

As with temperature, a slow shift in seawater pH is considered to be a potentially pervasive long-term threat to marine communities [11], [12]. There is concern at present about the gradually increasing acidity of our oceans driven by high, and increasing, levels of CO_2_ in our atmosphere [13], [14] and coastal eutrophication [15], [16]. Furthermore, it is also accepted that environmental stressors such as ocean acidification, eutrophication, and elevated temperature, among others, may interact synergistically in natural systems to affect biodiversity and ecosystem function [17], [18]. The biogeochemistry of coarse, permeable carbonate sands has been mainly investigated in tropical areas where carbonates from coral reefs comprise the majority of the sedimentary material [19], [20]. However, permeable carbonate sediments are also widely distributed in non-tropical regions [21], with shell hash from bivalves contributing the majority of the carbonate material.

There is evidence that carbonate systems play a key role in buffering ocean acidification [22]–[24]. However, CO_2_ exchange between the atmosphere and ocean is only partly controlled by carbonate mineral precipitation. Other major mechanisms in the system are production, respiration, remineralisation, and net sedimentary storage of organic matter in the ocean [25]. Therefore, to examine the potential for CaCO_3_ to alter biogeochemistry in temperate sediments, we performed an orthogonally crossed experiment involving additions of organic matter (OM) and CaCO_3_ (CC) to coarse, heterogeneous coastal sand (OM addition, CC addition, OM+CC addition as a mixture, and controls). Oxygen, dissolved inorganic carbon, and nutrient fluxes were measured in all treatments as response variables to understand effects on benthic respiration, net and gross primary production, and nutrient utilisation. Individually, these variables are key processes in benthic systems and commonly used indices of ecosystem functioning [26], [27]. However, we also examined changes in relationships between processes. Analysing the strength and variability of coupled processes like ammonium uptake and primary production, has been shown to be useful for assessing the impacts of subtle stressors in soft sediments [5]–[7], and this was the approach that we used here. We hypothesised that additions of CC would counteract those of OM, and that the treatment types would drive relationships in opposite directions.

## Materials and Methods

### Study site and sampling set up description

The study was conducted at a shallow soft-sediment site (9 m deep) with coarse permeable shelly sands and strong tidal currents in Kawau Bay (36° 23′ 55″ S, 174° 49′ 42″ E), a large embayment on the NE coast of North Island, New Zealand. This bay has a diversity of benthic soft-sediment habitat types [28] and the study site was particularly heterogeneous; that is, all the environmental variables measured exhibited a high degree of variability across the studied ecosystem (see Supporting Information, Figures S1 and S2). The area was dominated by an assemblage of large macrofauna, such as dog cockles (*Glycymeris glycymeris*), hermit crabs, sponges, shrimps (*Heterosquilla* sp.), fan worms (Serpulidae) as well as a variety of smaller organisms. All necessary permits were obtained for the described field studies. No specific permissions were required to study these locations, as they were away from shore (subtidal marine) and not within specially protected areas. The studies did not involve endangered or protected species, and the collection of cores for the study of macroinvertebrates was authorised by Special permits 505 and 542 from the Ministry for Primary Industries.

In November 2011, 48 square plots (50 cm×50 cm) were established over two days by scuba divers in a 12 column by 4 row array on the seafloor (length 30 m, width 8 m). Three treatment additions plus a control (n = 12 each) were interspersed in three identical blocks in a balanced crossed design (see Supporting Information, Figure S3). The treatments were created by adding substances to the sediment surface of the plots (0.25 m^2^): organic matter (OM; cornflour, 35 g.), calcium carbonate (CC; Aglime, 35 g.) or a mixture of both (Mix, 70 g total with equal parts OM and CC). The type and amount of OM used (food grade cornflour; aprox. 42% organic carbon, 0.1% organic nitrogen) was sufficient to elicit a benthic response but too little to cause sediment hypoxia or macrofaunal mortalities [5], [6]. Agricultural lime (McDonald's Aglime©; 94% CaCO_3_) is a natural product consisting of finely ground limestone. It is commonly used in agriculture to improve the quality of the soil by neutralising acid [29].

Each powdered substance was weighed into lidded containers and mixed with ambient seawater (100 mL) prior to application. This ensured that a slurry of material could be controllably added to the sediment surface. Square domes (50 cm×50 cm) were positioned atop the sediment surface and divers used syringes loaded with slurry to introduce the material to the plots (see Figure S3). The domes were left in place for 2–3 hours after treatment additions to allow the material to settle out of suspension and onto the sediment surface. After dome removal, a 1 mm mesh screen was positioned over the plots and ambient sediment was carefully sprinkled through the screen, thinly covering the treatment materials, reducing the likelihood of losses due to resuspension and ensuring incorporation into the sediment fabric. The experiment set up took over two days and left it for one week to allow the treatments to take effect.

### Measurements of oxygen and nutrient fluxes

A week after treatment establishment, benthic incubation chambers were deployed in the experimental plots. This interval provided time for the materials to be processed and to begin to affect porewater nutrient concentrations and sediment biogeochemistry. In particular, we expected that most of the organic material added to the plots would be consumed and processed by the biota within a week, approximately, due to the relatively small amount of organic material added and the high activity rates in these types of ecosystems [5], [6], [30].

Three consecutive days were required to sample all 48 plots, with 16 chambers (4 replicates of each treatment) deployed per day. We were able to block the plots by establishment date (the eastern half of the array was established on day 1, the western half on day 2) or by sampling date (the eastern third was sampled on day 8, the middle third on day 9, the western third on day 10). The chambers were used to assess fluxes of oxygen and nutrients across the sediment-water interface. During deployment, a square chamber base (an aluminium border with sides 50×50×10 cm tall) was positioned around the perimeter of each experimental plot and pressed halfway down into the sediment. Clear acrylic plastic lids were fitted to the chamber bases and clamped in place, isolating a square patch of sediment and approximately 25 L of overlying seawater for incubation. Each chamber contained one D-Opto dissolved oxygen and water temperature logger (60 data points per hour) and a Seabird electronic pump (20 ml/sec flow rate). The pump ran for 5 seconds every 45 seconds to provide intermittent non-directional stirring of chamber waters, preventing stagnation and the build-up of boundary layer gradients.

The seawater enclosed within each chamber was collected through syringe activated sampling ports at the beginning and end of each incubation period (∼4 hours). Collection times were recorded in all instances. Incubations were run during daytime: once with photosynthesis possible (clear chamber lids in the morning between 0800 and 1230 hrs); and once without photosynthesis (black chamber covers in the afternoons between 1230 and 1750). Prior to every incubation period (including in between each day's light and dark incubations), all of the chambers were flushed with ambient bottom water by briefly removing the chamber lids; this ensured that all chamber incubations were initialised (once lids were replaced) with ambient bottom water unaffected by previous runs. As biological reactions are often light and temperature dependent, we assessed underwater light levels and temperatures with submersible Hobo® loggers deployed to 10 of the 16 chambers (5 cm above the sediment surface, logging every 5 min) on each of the 3 days of sampling.

As soon as water samples (4 replicates per treatment, 48 samples total) were back aboard the boat, levels of dissolved oxygen (Hach® HQ40d dissolved oxygen sensor, accuracy ±0.1 mg/L DO) and pH (PreSense® needle-type microsensor, accuracy ±0.05 pH units) were measured. Water samples were then filtered (1.1 µm Whatman filter) and stored frozen and in the dark until analysis for dissolved inorganic nutrient concentrations. Samples for dissolved inorganic carbon (DIC) were filtered and preserved in saturated mercuric chloride in 100 mL glassware prior to analysis.

### Sediment characteristics

Upon completion of the chamber incubations, the chamber lids were removed and two small cores of surface sediment (5 cm^2^×2 cm deep) were collected from every plot for chlorophyll *a*, organic matter and grain size analyses. After collection, all samples were frozen and stored in dark. In addition, one macrofaunal core (133 cm^2^×15 cm deep) from the middle of each plot was collected, sieved across a 0.5 mm mesh and preserved in 70% IPA. Lastly, the remaining area of sediment inside each plot was searched and data on the abundance of large organisms (e.g., dog cockles, burrowing urchins, sponges, shrimp burrows) was collected.

### Laboratory analysis

Fluxes of dissolved oxygen and inorganic nutrients (*F*) were calculated in all light and dark chambers based on the magnitude of change in water chemistry during incubation,
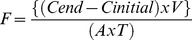
where *C* is nutrient or oxygen concentration (µmol l^−1^) at the beginning or end of the incubation, *V* is the volume of seawater inside the chamber (25 l), *A* is the area of sediment enclosed by the chamber (0.25 m^2^), and *T* is the elapsed time between initial and final samplings (hours).

Fluxes of DO in light chambers provided an estimate of net primary production (NPP); while fluxes of DO in dark chambers were used to estimate benthic community respiration (CR). Gross primary production (GPP) was calculated by subtracting CR from NPP. Analysis for ammonium nitrogen (NH_4_^+^- N), nitrate-plus-nitrite nitrogen (NO_X_-N) and reactive phosphate phosphorus (HPO_4_^2−^-P) were performed using standard methods [31] on an Astoria Pacific International autoanalyzer (Clackamas, Oregon, USA) with detection limits of <1 µmol/L for N and P. Dissolved inorganic carbon (DIC) was obtained by means of a total organic carbon analyser (APHA5310C method) with detection limits of <0.2 µmol/L for C. NH_4_^+^- N uptake was calculated as dark NH_4_^+^-N flux minus light NH_4_^+^-N flux. NO_X_-N and HPO_4_^2−^P uptakes were similarly calculated. Dissolved inorganic nitrogen (DIN) is sum of dissolved NH_4_^+^-N and the NO_X_-N.

For sediment analysis, grain size samples were thawed and homogenized, and 5 g subsamples were digested in H_2_O_2_ until frothing ceased. The sediment was then wet sieved through 2000, 500, 250 and 63 µm mesh sieves. All fractions were dried at 60°C and weighed after 2 days. Sediment chlorophyll *a* (chla) content (µg.g^−1^ sediment) was used as an indicator of microphytobenthos abundance. Chla samples were freeze-dried prior to analysis (to standardize sample water content) and then homogenized. Subsamples (5 g) were boiled in 90% ethanol to extract the photopigments that were then analysed spectrophotometrically. An acidification step was used to separate degradation products from chla [32]. Organic matter content samples were thawed and homogenized, and 5 g subsamples were dried at 60°C. Dry masses were obtained after 2 days, and samples were moved to a 400°C combustion oven (6 hours). Organic matter content was assessed as the sediment's percentage mass lost during combustion.

Macrofauna were sorted and identified to the lowest taxonomic grouping practicable. From the raw data, we calculated the total abundance, taxonomic richness, and diversity of macrofauna in every plot. We later classified the macrofauna into main feeding guilds (deposit feeders, suspension feeders, predators/scavengers).

### Data analysis

Patterns in the data were first assessed with boxplots, Cleveland dotplots, and pair plots to identify outliers and the potential for collinearity among explanatory variables. Sediment characteristics and macrofauna communities were then assessed by means of analysis of variance (ANOVA). Treatment and block were included in two-way ANOVA models; treatment was used as a fixed factor with four levels (organic matter, calcium carbonate, mixture and control) and “block” was included to test for the influence of either establishment date (2 levels: eastern and western halves of the sampling array) or sampling date (3 levels: eastern, middle and western thirds of the sampling array) (see Supporting Figure S3). Neither type of blocking had significant interaction effects with the treatments in the two-way ANOVA models (see Supporting information, Table S1), so block was removed as a predictor variable from all subsequent analyses.

The response variables of interest (fluxes) and the explanatory co-variables (sediment characteristics, macrofauna, etc.) both exhibited a high degree of variability, reflecting the heterogeneity of the habitat (see Figures S1 and S2, Supporting Information). Also, as we were interested in relationships between processes, a continuous approach (e.g., regression based models or analysis of co-variance based) was more appropriate for this type of than a purely categorical approach. Therefore, we used generalized linear models (GLMs: Gaussian distribution, identity link function) to test for significant interactions between explanatory variables and treatments. Since three days were required to sample all the plots, the relationships between the main variables and treatment effects may have been influenced by the different sampling dates. Therefore, we included sampling date (3 days, one block each day) as a factor in the models. Unsurprisingly, there were some significant differences over time (see Supporting Information, Table S2). However, no significant interactions between sampling date and treatment and/or among sampling date, treatment and the main variables were found (Table S2), so time was dropped from the final models and treatment remained as the only factor. Finally, we ran the models first without treatment and later including treatment as a fixed factor to find those predictors mediated by the treatments. *A posteriori* contrasts were performed by reordering the levels of a factor to represent the effect of the treatments.

Akaike's Information Criterion (AIC) and the proportional increase in explained deviance (pseudo R^2^; [33]) were used to evaluate each model fit and parsimony. In general, a variable was retained in the model only if it caused a significant increase in deviance when it was removed from the current model. Model assumptions were checked using (i) plots of residuals versus fitted values to verify homogeneity, (ii) quantile-quantile plots or histograms of the residuals for the assessment of normality, and (iii) residuals versus each explanatory variable to check for data independence. All the analyses were fitted in R 2.15.1 [34], glm package.

## Results

**Figure 1:**
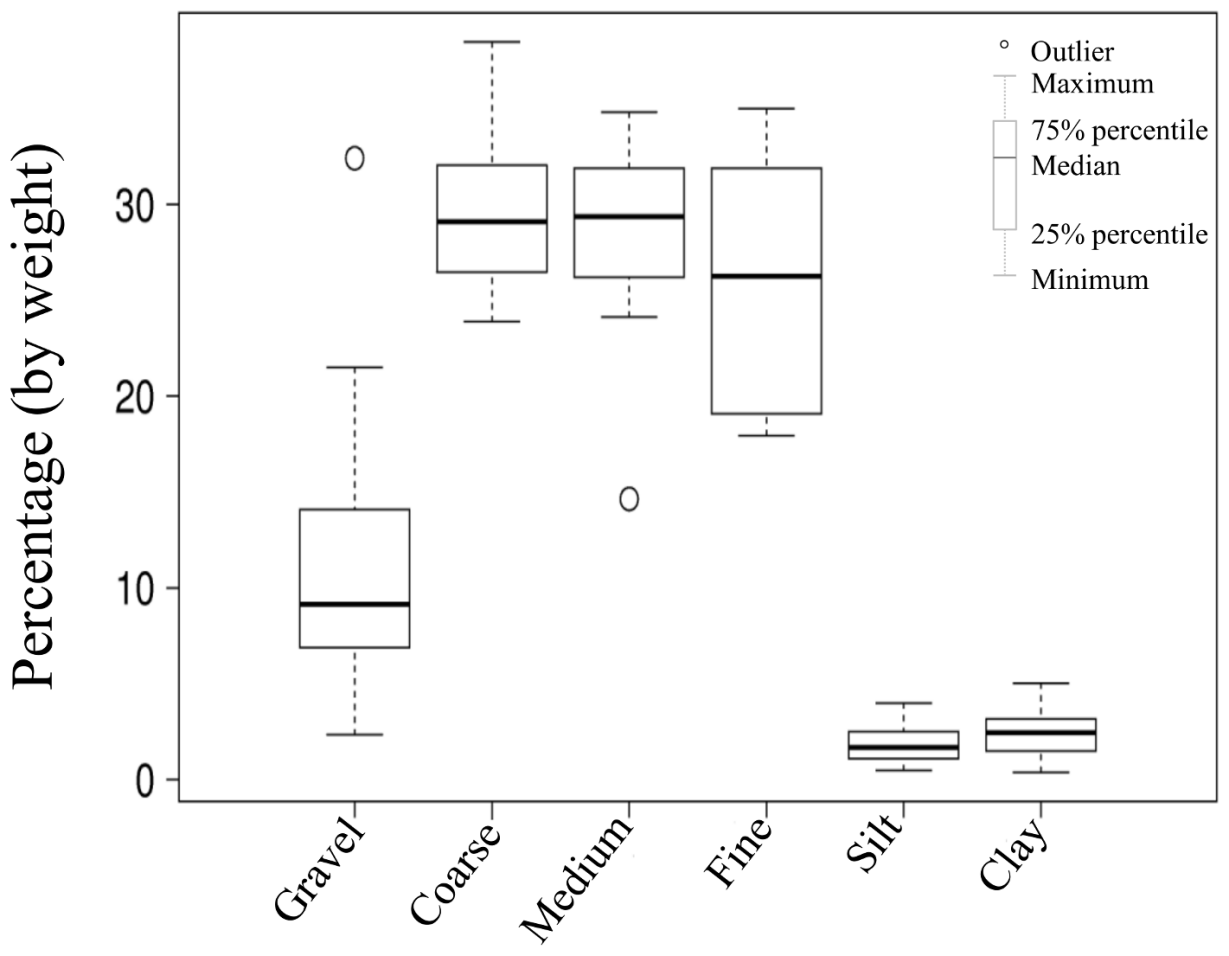
Boxplots showing the interquartile range (first quartile, median and third quartile) of the sediment data. Whiskers: the values that extend to 1.5 times the interquartile range. When there are no outliers the whiskers show the maximum and minimum values.

Sediments at the sampling site were heterogeneous, with almost even mixtures of fine + very fine sand (63–250 µm, 26% by weight), medium sand (250–500 µm, 28.2%) and coarse + very coarse sand (500–2000 µm, 29.7%) (Figure 1). Gravel content (>2000 µm) was approximately 12%, with the large particles mostly comprised of bivalve shell fragments. Mud (<63 µm) was 4.2% of the total sedimentary composition (Figure 1).

There was a high degree of variability in the response variables that we measured, likely reflected the heterogeneity of the habitat (see Figures S1 and S2, Supporting Information). Sediment chlorophyll *a* (µg.g^−1^ sediment) and organic matter content (%) showed high variability (see Figure S1), and no significant differences were detected among the treatments (see Supporting Information, Table S1). Chla ranged between 2 to 4 µg.g^−1^ (IQR), and organic matter content between 2 to 3% (IQR) (Figure S1). Organic matter changed significantly (p<0.01) during the sampling dates, but no significant interaction was found between treatments (Table S1).

The abundance of large macrofaunal organisms was distributed haphazardly with respect to treatments at the site (Figure S1), with no significant differences detected among treatments (see Table S1 for the ANOVA summary). The rest of the macrofauna community components (total macrofauna abundance, species richness and the abundance of deposit and suspension feeders) also showed a variable distribution, again with no significant differences among treatments detected (see Table S1 and Figure S1, Supporting Information).

Water samples showed a pH range between 8.418 and 8.523 pH units (8.479±0.030). Statistical results indicated a significant change in pH during the sampling dates (p<0.001), but no significant interaction was found between treatments (see Table S1). Light intensity (F_2,24_ = 158.98) and temperature (F_2,24_ = 48.3) at the seabed changed significantly (p<0.001) during the 3 sampling days. However, light (F_3,23_ = 0.095, p = 0.962) and temperature (F_3,23_ = 0.614, p = 0.613) were not significantly different among treatments during this period.

**Figure 2:**
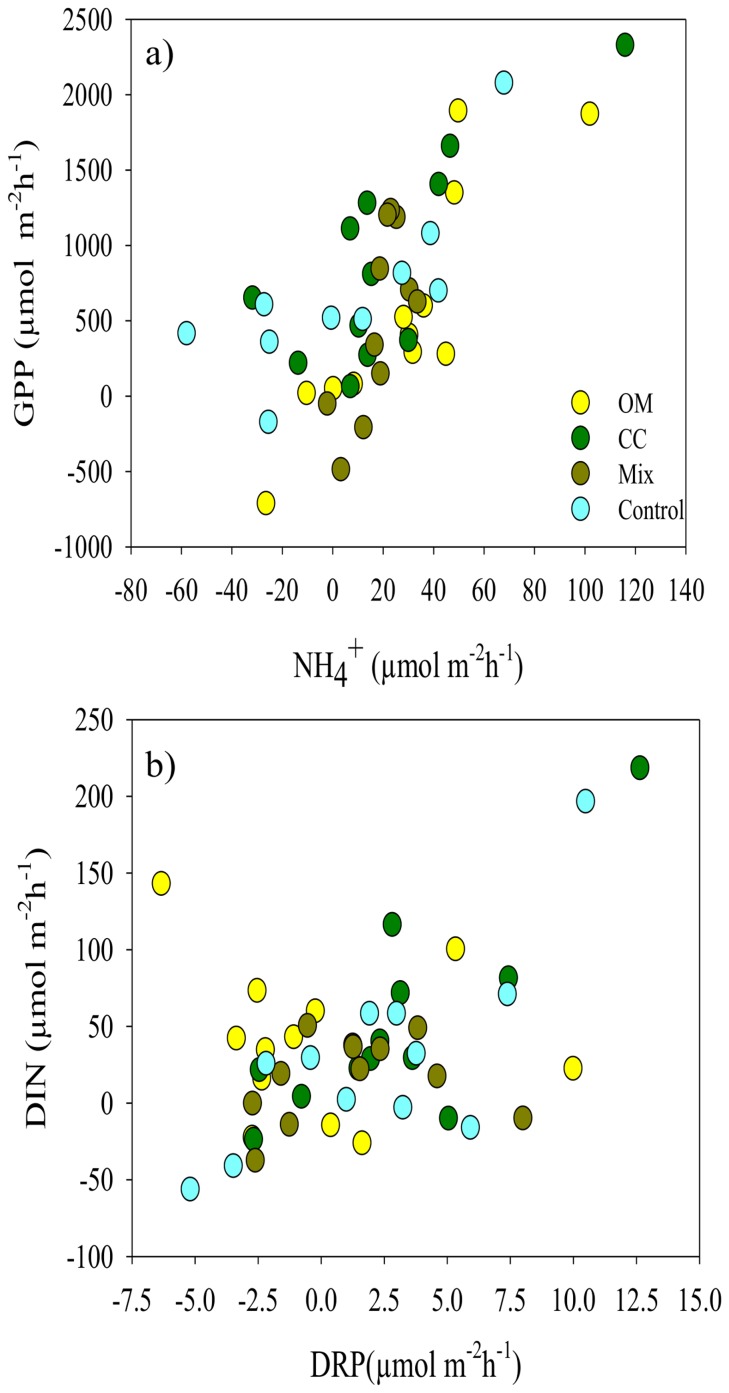
Relationship between main biogeochemical coupled processes (raw data). The top panel (a) shows the general relationship between gross primary production (GPP, µmol O_2_ m^−2^h^−1^) and ammonium uptake (µmol NH_4_^+^-N m^−2^h^−1^) across the sampling area. The bottom panel (b) shows the relationship between dissolved inorganic nitrogen uptake (µmol DIN m^−2^h^−1^) and dissolved reactive phosphorus (µmol DRP m^−2^h^−1^) across the sampling area. Treatments: Organic Matter, Calcium carbonate, Mix, Control.

The most parsimonious models (Models 1 to 4) relating different environmental and macrofauna community variables, first without treatment (a) and later including treatment (b) as a factor are presented in Table 1. Dissolved inorganic carbon in the presence of light (DIC_light_), rates of NH_4_^+^ and NO_3_^−^ uptake and the abundance of deposit feeders (DF) were significant predictors of benthic gross primary production (GPP) at our site in Kawau Bay (Model 1a), irrespective of treatment (Table 1). However, when treatment was included as a factor in the model, the only predictor variable showing a significant effect was NH_4_^+^ uptake (Table 1, Model 1b). NH_4_^+^ uptake showed a significant positive relationship with GPP (Figure 2a). There was no significant interaction between the response variable and treatment (NH_4_^+^*Tr) in this model (Model 1b; F_1,3_ = 2.18, p = 0.106). Therefore, we dropped the interaction term from the analysis and ran the model again (final Model 1b, Table 1). Our analysis revealed a significant effect of treatment on the form of the NH_4_^+^–GPP relationship (see Table 2). OM and Mix plots produced significantly stronger NH_4_^+^–GPP relationships than Control and CC plots (Table 2, Figure 2a). Significant differences were observed between treatment pairs, except for between Mix and OM, and C and CC. The GLM summary for all the treatments is presented as supporting information (see Table S3).

**Table 1:** The most parsimonious Generalized Linear Models (GLMs) relating different environmental and macrofauna community variables with and without treatment as a factor (terms that were not significant were dropped from the final models).

Model name	Model expression (without treatment)	AIC	pseudo-R^2^	Model name	Model expression with treatment interaction (*Tr)	AIC	pseudo-R^2^
Model 1a	GPP∼DIC_light_+NH_4_^+^+NO_3_^−^+DF	733.9	0.609	Model 1b§	GPP∼NH_4_^+^+NH_4_^+^*Tr	682.1	0.655
Model 2a	DIN∼GPP+DRP+DF	434.1	0.699	Model 2b	DIN∼DRP+DRP*Tr	464.9	0.493
Model 3a	Chla∼Abundance	135.1	0.100	Model 3b	Chla∼Abundance+Abundance*Tr	134.12	0.334
Model 4a	NO_3_^−^_light_∼DF	387.8	0.100	Model 4b	NO_3_^−^_light_∼DF+DF*Tr	388.8	0.277

The Akaike's Information Criterion (AIC) and the proportional increase in explained deviance (pseudo-R^2^) were used to evaluate each regression-based model fit and parsimony.

NH_4_^+^: ammonium uptake; NO_3_^−^: nitrate uptake; DIN: dissolved inorganic nitrogen (Σ NH_4_^+^+NO_3_^−^); NO_3_^−^_light_: nitrate flux during daylight.

DRP: dissolved reactive phosphorus (HPO_4_^2−^- P); DIC_light_: dissolved inorganic carbon flux during daylight.

GPP: gross primary production; Chla: chlorophyll *a* concentration.

Abundance: total macrofauna abundance; DF: deposit feeders abundance.

pseudo-R^2^ = (null deviance-residual deviance)/null deviance; (*sensu* [46]).

§ There was no significant interaction between the response variable and treatment (NH_4_^+^*Tr). We dropped the interaction term from the analysis and ran the model again. Final Model 1b: GPP∼NH_4_^+^+Tr (AIC = 683.4, pseudo-R^2^ = 0.593).

**Table 2:** ANOVA output from the GLM Model 1b from Table 1 (Gaussian distribution, identity link function) indicating the significance of ammonium uptake (NH_4_^+^) on gross primary production (GPP) among treatments (OM: Organic matter, CC: Calcium carbonate, Mix: OM+CC, and C: Control).

ANOVA output: GPP∼NH_4_^+^+ Treatment					Treatment Contrasts (*p* results)				
Source	Rs df	Rs Dev	F	*p*	Tr	C	CC	Mix	OM
Null	44	19561863			C	-			
NH_4_^+^	43	9960327	48.3	*<0.001* ***	CC	ns	-		
Treatment (Tr)	40	7951120	3.37	0.027*	Mix	***	*	-	
					OM	***	*	ns	-

No significant interaction was found (NH_4_^+^*Tr; F_1,3_ = 2.18, p = 0.106). The Treatment effect (p<0.05) sizes are showed as contrasts (see summary of the full regression-based model in Table S3).

ns: *p*>0.1,

+ 0.05<*p*<0.1,

*p<0.05,

**p<0.01,

***p<0.001.

Rs df: residual degrees of freedom; Rs Dev: residual deviance.

Gross primary production (GPP), dissolved reactive phosphorus flux (DRP) and the abundance of deposit feeders (DF) were significant predictors of DIN flux irrespective of treatment (Table 1, Model 2a). However, when treatment was included in the model, only DRP remained as a significant predictor variable (Table 1, Model 2b). The relationship between fluxes of DIN and DRP was positive overall (Figure 2b), though this relationship became negative as a result of the OM treatment (Figure 2b), signalling a significant interaction between DRP and treatment type in the prediction of DIN flux (Table 3). Judging from *post hoc* treatment contrasts, the form of the DRP–DIN flux relationship differed between OM plots and both Control and CC plots (Table 3). The GLM summary for all the treatments is presented as supporting information (see Table S4).

**Table 3:** ANOVA output from the GLM Model 2b (Gaussian distribution, identity link function) indicating the significance of dissolved reactive phosphorus (DRP) on dissolved inorganic nitrogen (DIN) among treatments (OM: Organic matter, CC: Calcium carbonate, Mix: OM+CC, and C: Control).

ANOVA output (GLM: DIN∼DRP*Treatment)					Contrasts (*p* results)				
Source	Rs df	Rs Dev	F	*p*	DRP*Tr	C	CC	Mix	OM
Null	43	131192			C	-			
DRP	42	99762	17.01	*<0.001* ***	CC	ns	-		
Treatment (Tr)	39	95093	0.84	0.479	Mix	ns	ns	-	
DRP*Tr	36	66519	5.15	0.005**	OM	**	***	ns	-

The DRP*Treatment (p<0.01) interaction effects are showed as contrasts (see summary of the full regression-based model in Table S4).

ns: *p*>0.1,

+ 0.05<*p*<0.1,

*p<0.05,

**p<0.01,

***p<0.001.

Rs df: residual degrees of freedom; Rs Dev: residual deviance.

**Figure 3:**
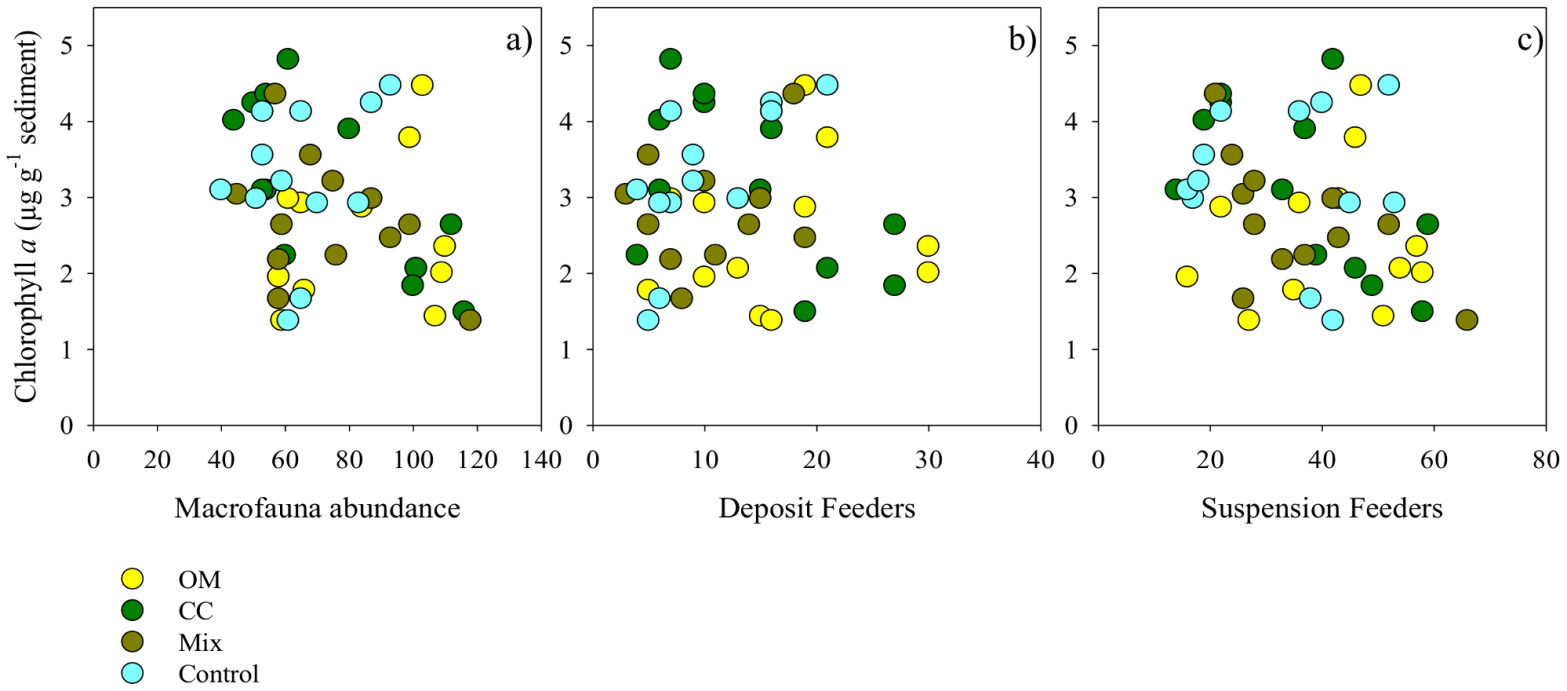
Biplots showing the relationship between chlorophyll *a* vs. macrofauna abundance, and the main feeding guilds. The panels show the general relationship across the sampling area. Chlorophyll *a* (µg g^−1^ sediment), total macrofauna abundance (number of individuals), main feeding guilds (abundance of deposit and suspension feeders; DF and SF, respectively). Plots are raw data. (Treatments: Organic Matter, Calcium carbonate, Mix, Control).

Macrofauna abundance was a significant predictor of chlorophyll *a* (Table 1, Model 3). The overall relationship, although negative and significant (Table 4), was not visually clear (Figure 3a). This relationship was mainly defined by the abundance*treatment interaction (Abund*Tr, Table 4). The chla vs. abundance relationship was significantly different in CC plots relative to OM and Control plots (Table 4). In the OM and Control plots this relationship was positive, whereas it was negative in the treatments containing calcium carbonate (Mix and CC) (Figure 3a). There was also a significant and negative relationship between chla and the abundance of deposit feeders (DF, Figure 3b) mainly defined by the DF*treatment interaction (DF*Tr, Table 4). Again, this relationship differed in plots with and without calcium carbonate: negative in CC and Mix treatments, positive in OM and Control plots (Table 4, Figure 3b). The overall relationship between chla and the abundance of suspension feeders (SF) was also significant and negative (F_1,46_ = 5.82; p = 0.021), but no changes in the relationship according to treatment type were detected (F_3,40_ = 1.76; p = 0.171; Figure 3c). The GLM summary for all the treatments is shown as supporting information (see Table S5).

**Table 4:** ANOVA output from the GLM Model 3b (Gaussian distribution, identity link function) indicating the significance of chlorophyll *a* content on the total macrofauna abundance and the abundance of deposit feeders (DF) among treatments (OM: Organic matter, CC: Calcium carbonate, Mix: OM+CC, and C: Control).

ANOVA output (GLM: Chla∼Abundance*Treatment)					Abundance*Tr Contrasts (*p* results)				
Source	Rs df	Rs Dev	F	*p*	Tr	C	CC	Mix	OM
Null	47	45.44			C	-			
Abundance	43	41.4	5.12	*0.03* *	CC	*	-		
Treatment (Tr)	43	38.7	1.2	0.353	Mix	ns	ns	-	
Abund*Tr	40	31.6	3.02	*0.04* *	OM	ns	*	ns	-

ANOVA output (GLM:Chla∼DF*Treatment)					DF*Tr Contrasts (*p* results)				
Source	Rs df	Rs Dev	F	*p*	Tr	C	CC	Mix	OM
Null	46	43.1			C	-			
DF	45	42.9	0.19	0.658	CC	***	-		
Treatment (Tr)	42	39.1	1.71	0.18	Mix	ns	ns	-	
DF*Tr	39	29.3	4.37	*0.009* **	OM	+	*	ns	-

The Abundance*Treatment (p<0.05) and DF*treatment (p<0.01) interaction effects are showed as contrasts (see summary of the full regression-based model in Table S5).

ns: *p*>0.1,

+ 0.05<*p*<0.1,

*p<0.05,

**p<0.01,

***p<0.001.

Rs df: residual degrees of freedom; Rs Dev: residual deviance.

**Figure 4:**
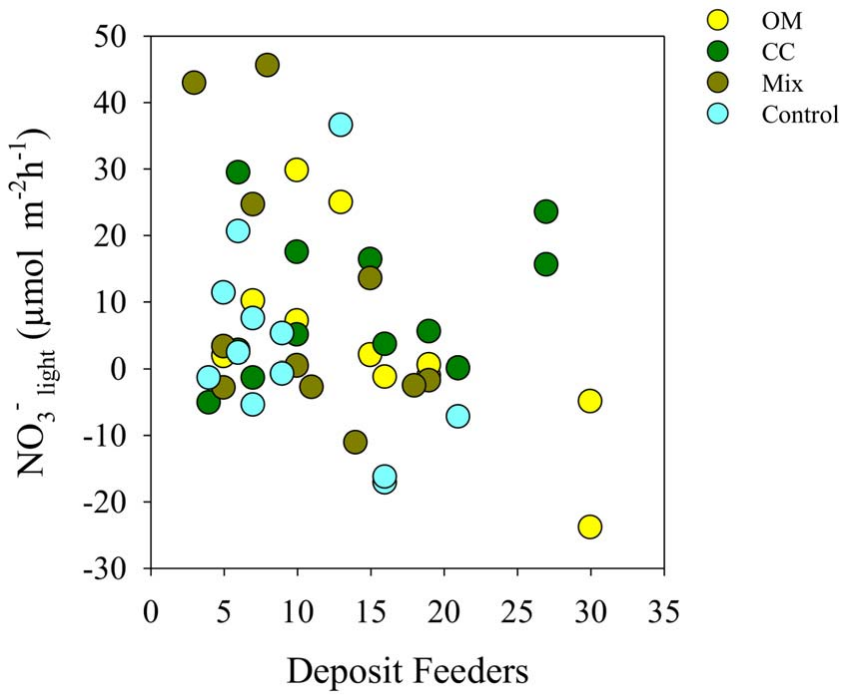
Relationship between nitrate flux during daylight (µmol NO_3_^−^_light_ m^−2^h^−1^) and the abundance of deposit feeders. The panel shows the general relationship across the sampling area. Plots are raw data. (Treatments: Organic Matter, Calcium carbonate, Mix, Control).

The abundance of deposit feeders was a significant predictor of light nitrate flux (Table 1, Model 4); the relationship was negative (Table 5, Figure 4). The relationship changed marginally (p<0.10) according to treatment, differing in the CC treatment relative to the OM and Mix treatments (Table 5, Contrasts). The estimated regression parameter was positive for CC and negative for OM and Mix (Figure 4). The GLM summary is given as supporting information (see Table S6).

**Table 5:** ANOVA output from the GLM Model 4b (Gaussian distribution, identity link function) indicating the significance of NO_3_^−^ flux during daylight on the abundance of deposit feeders (DF) among treatments (OM: Organic matter, CC: Calcium carbonate, Mix: OM+CC, and C: Control).

ANOVA output: NO_3_^−^_light_∼DF*Treatment					DF*Tr Contrasts (*p* results)				
Source	Rs df	Rs Dev	F	*p*	Tr	C	CC	Mix	OM
Null	46	10145.6			C	-			
DF	45	9271.6	4.65	*0.03* *	CC	ns	-		
Treatment (Tr)	42	8716.1	0.98	0.411	Mix	ns	*	-	
DF*Tr	39	7343.5	2.43	0.07+	OM	ns	*	ns	-

The DF*Treatment interaction (p = 0.07) effect is showed as contrasts (summary of the full regression-based model in Table S6).

ns: *p*>0.1,

+ 0.05<p<0.1,

*p<0.05,

**p<0.01,

***p<0.001.

Rs df: residual degrees of freedom; Rs Dev: residual deviance.

## Discussion

In marine soft-sediment systems, subtle changes to sediment characteristics have been shown to alter relationships between biogeochemical processes (e.g. nutrient utilisation and primary production [5], [6]), suggesting that an analysis of coupled relationships may be appropriate for tracking the effects of subtle sublethal stressors.

In this study, we examined the response of a heterogeneous soft-sediment subtidal system to subtle, experimentally controlled, changes in sediment organic matter and calcium carbonate content. Organic matter occurs naturally in all sediments, with stocks of organic matter dependent upon the relative balance of supply (e.g. from the water column above) and degradation by organisms living in the sediment. The amount of organic matter in the sediment tends to be highest in eutrophic areas, with supply outpacing degradation. Organic enrichment of sediments has predictable consequences on sediment biogeochemistry and macrofaunal community composition [35]. For example, organic matter mineralization results in the formation of organic and inorganic acids, associated with O_2_ consumption, which causes the pH of coastal bottom waters to decline [16], [36]. However, most of the conceptual models for organic enrichment are designed to explain changes across very steep organic matter gradients.

Calcium carbonate is another naturally occurring substance in coastal sediments. Calcium carbonate concentrations are particularly high in tropical coral sands, but can be high in shell dominated sands in the middle and higher latitudes as well [21]. Whilst organic enrichment will result in increased rates of benthic respiration, elevated pCO_2_ and increased porewater acidity, calcium carbonate is thought to counter these effects, acting as a buffer, though our knowledge of how (and whether) this occurs remains poorly understood [15], [16].

In this study, we examined relationships between key biogeochemical and sedimentary variables to understand the individual and interactive effects of OM and CC addition. For example, we assessed the strength and variability of relationships between coupled processes such as gross primary production and ammonium release [6], [7]. We expected primary production to be positively related to NH_4_^+^ uptake (with microphytes taking up this form of nitrogen in order to meet the demands of photosynthesis) and suggested the breakdown of this relationship in response to OM or CC additions to be a signal of impaired functioning. Furthermore, it is well known that stressors or responses such as abundance/diversity act in combination in natural systems, not in isolation. The interactive effects of multiple stressors on the ecosystem are more difficult to understand and predict [37]–[39]. For instance, in our study, the interaction between treatment and macrofauna abundance affected total chlorophyll *a* significantly; even though, according to our model (Table 4), treatment as a single variable was not significant. Therefore, the interactions may play a greater role in determining how an ecosystem responds than individual stressors, and at the same time those stressors may interact with other factors, such as abundance.

The present experiment showed that the additions tended to act in opposite directions, as we hypothesized, and demonstrated that highly heterogeneous sedimentary habitats can be surprisingly sensitive to subtle perturbations. For instance, fluxes of NH_4_^+^ were affected by the experimental manipulation. It is known that the decomposition and remineralisation of OM in sediments releases nutrients to the overlying water [40], and that the first form of DIN to appear during OM remineralisation is NH_4_^+^. This essential nutrient is also readily utilised by bacteria and algae and has a positive effect on primary production in systems where nitrogen is limiting [41], [42]. Gross primary production (GPP) was significantly higher in OM and Mix plots during this experiment when rates of NH_4_^+^ uptake were elevated, and this increase was significantly higher than in CC or Controls. We also documented changes in the relationship between N and P fluxes following treatment application, with a marked increase in nitrogen (DIN) to phosphorus (DRP) flux ratios in the OM plots. Regeneration of both N and P is expected to occur during the remineralisation of OM [43], generating the positive relationship between the two variables.

Although the application of organic matter and calcium carbonate material to the plots affected relationships between macrofauna and sediment biogeochemistry, no significant changes in macrofauna community characteristics (i.e., macrofauna abundance, diversity, number of species, feeding guilds) occurred among plots. However, macrofauna abundance was correlated with variables including nutrient fluxes and primary production. Specifically, the abundance of deposit feeders showed a negative relationship with NO_3_^−^ flux during daylight in Control and OM plots, but the relationship changed to positive when calcium carbonate was added to the CC plots. Similarly, the relationship between macrofauna abundance (and deposit feeders) and chlorophyll *a* was positive in OM plots, but became negative in plots with both CC and Mix. The DIN that is regenerated during OM decomposition is likely available for use by microphytobenthos, the abundance of which can be inferred from chla. Moreover, an increase in the OM decomposition will tend to promote an increase in sediment oxygen demand, as bacteria and macrofauna utilise this source of carbon. A reduced availability of O_2_, and an excess of CO_2_, will reduce nitrification rates and therefore NO_x_^−^ efflux in OM plots [44], [45]. Thus, OM additions can conceivably account for an increase in NH_4_^+^ and chla in the treated plots and the negative effects on nitrate flux. CC additions would tend to counteract these effects.

Most of the seafloor is composed of unconsolidated sediments, and considerable biodiversity exists in these types of systems [46], [47]. Most of the studies of carbonate sediments have focused on coral sands in tropical areas, and there is little information on the influence of carbonate materials on nutrient fluxes and biologically mediated ecosystem functions. The role of carbonates is increasingly important to define, given the steady rise in atmospheric CO_2_ concentration and coastal eutrophication, with resultant acidification of seawater. CO_2_ is sequestered in the form of CaCO_3_ by calcifying species, and this remains in the hard shells in the sediment long after the organisms have died [48]–[50]. Understanding what the changes in sediment calcium carbonate concentration mean for marine ecosystem functions is pivotal in a world with rising atmospheric CO_2_ concentrations [13].

The present contribution points out the high sensitivity of a highly heterogeneous soft-sediment ecosystem to small changes in biogeochemical drivers. Our study suggests that sediment carbonates may play a role in partially ameliorating coastal eutrophication effects by counteracting the effects of sediment organic matter enrichment. By analysing shifts in coupled relationships (*sensu* [6]) we were able to detect changes in the functioning of heterogeneous shallow marine carbonate sands at very low magnitudes of disturbance. This is useful, as predicting an ecosystem response prior to large and potentially irreversible changes is the key to the sustainability of our natural resources.

## Supporting Information

Figure S1 **Boxplots on sediment characteristics: chlorophyll a (µg.g-1 sediment), organic matter (%), and macrofauna community abundance (number of individuals) and Shannon's diversity index (H′) through the four treatments (Calcium carbonate, Control, Mix and Organic matter).**
(TIF)

Figure S2 **Boxplots showing the variability of the oxygen and nutrient fluxes through the four treatments (Calcium carbonate, Control, Mix and Organic matter).** Gross primary production (GPP), net primary production (NPP), ammonium uptake (NH_4_^+^), dissolved reactive phosphorus (DRP), dissolved inorganic nitrogen uptake (DIN_uptake_), dissolved inorganic nitrogen in daylight (DIN_light_), nitrate uptake and during daylight (NO_3_^−^_uptake_ and NO_3_^−^_light_), dissolved inorganic carbon during daylight (DIC_light_). Units: µmol m^−2^ h^−1^.
(TIF)

Figure S3 **Schematic diagram showing the layout of the plots with the treatments (Calcium carbonate, Organic matter, Mix: OM+CC, and Control) across the sampling area in a balanced orthogonal design with randomized interspersed treatment positions (3 blocks).** Below a picture showing a diver introducing the treatment inside one of the plots through a syringe activated sampling port. Plots were made of two parts: a square chamber base (aluminum border with sides 50×50 cm×10 cm tall) pressed down into the sediment (∼5 cm) and a clear acrylic plastic dome (50×50 cm) fitted to the chamber bases and clamped in place.
(TIF)

Table S1 **Summary of the ANOVAs (Treatment and blocks as fixed factors) for sediment characteristics (chlorophyll a and organic matter), water pH and macrofauna communities (Abundance of large organisms, total abundance, number of species and the abundance of deposit and suspension feeders).** Treatments: OM: Organic matter, CC: Calcium carbonate, Mix: OM+CC, Control. Significant results in italics.
(DOCX)

Table S2 **Summary of the ANOVA outputs from the Generalized Linear Models (** **Table 1** **), including Treatment and Day (3 sampling days) as fixed factors.**
(DOCX)

Table S3 **Generalized Linear Model summary (regression-based models with Gaussian distribution and identity link function) indicating the significance of ammonium uptake (NH_4_^+^) on gross primary production (GPP) among treatments (OM: Organic matter, CC: Calcium carbonate, Mix: OM+CC, Control).**
(DOCX)

Table S4 **Generalized Linear Model summary (regression-based models with Gaussian distribution and identity link function) indicating the significance of dissolved reactive phosphorus (DRP) on dissolved inorganic nitrogen (DIN) among treatments (OM: Organic matter, CC: Calcium carbonate, Mix: OM+CC, Control).**
(DOCX)

Table S5 **Generalized Linear Model summary (regression-based models with Gaussian distribution and identity link function) indicating the significance of chlorophyll** ***a*** **content on the total macrofauna abundance and the abundance of deposit feeders among treatments (OM: Organic matter, CC: Calcium carbonate, Mix: OM+CC, Control).** SE: Standard Error.
(DOCX)

Table S6 **Generalized Linear Model summary (regression-based models with Gaussian distribution and identity link function) indicating the significance of NO_3_^−^ flux during daylight on the abundance of deposit feeders (DF) among treatments (OM: Organic matter, CC: Calcium carbonate, Mix: OM+CC, Control).**
(DOCX)
