# Supplementary figures and images for: Sensitivity of Heterogeneous Marine Benthic Habitats to Subtle Stressors
Source: PLoS One. 2013 Nov 28;8(11):e81646. doi: 10.1371/journal.pone.0081646 (PMC3842950; doi:10.1371/journal.pone.0081646)

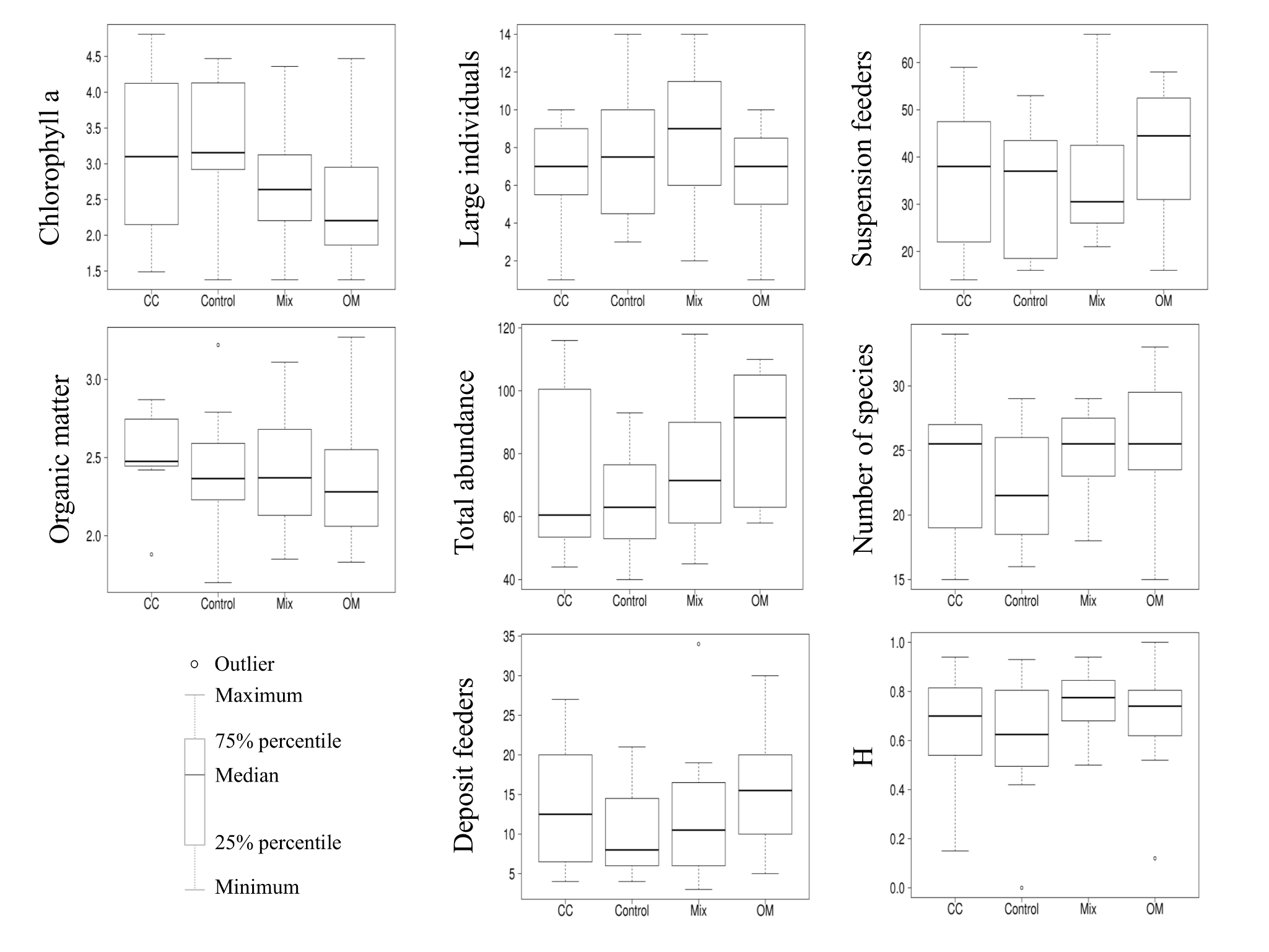

Supplement: Figure S1 — Boxplots on sediment characteristics: chlorophyll a (µg.g-1 sediment), organic matter (%), and macrofauna community abundance (number of individuals) and Shannon's diversity index (H′) through the four treatments (Calcium carbonate, Control, Mix and Organic matter). (TIF) [file pone.0081646.s001.tif]

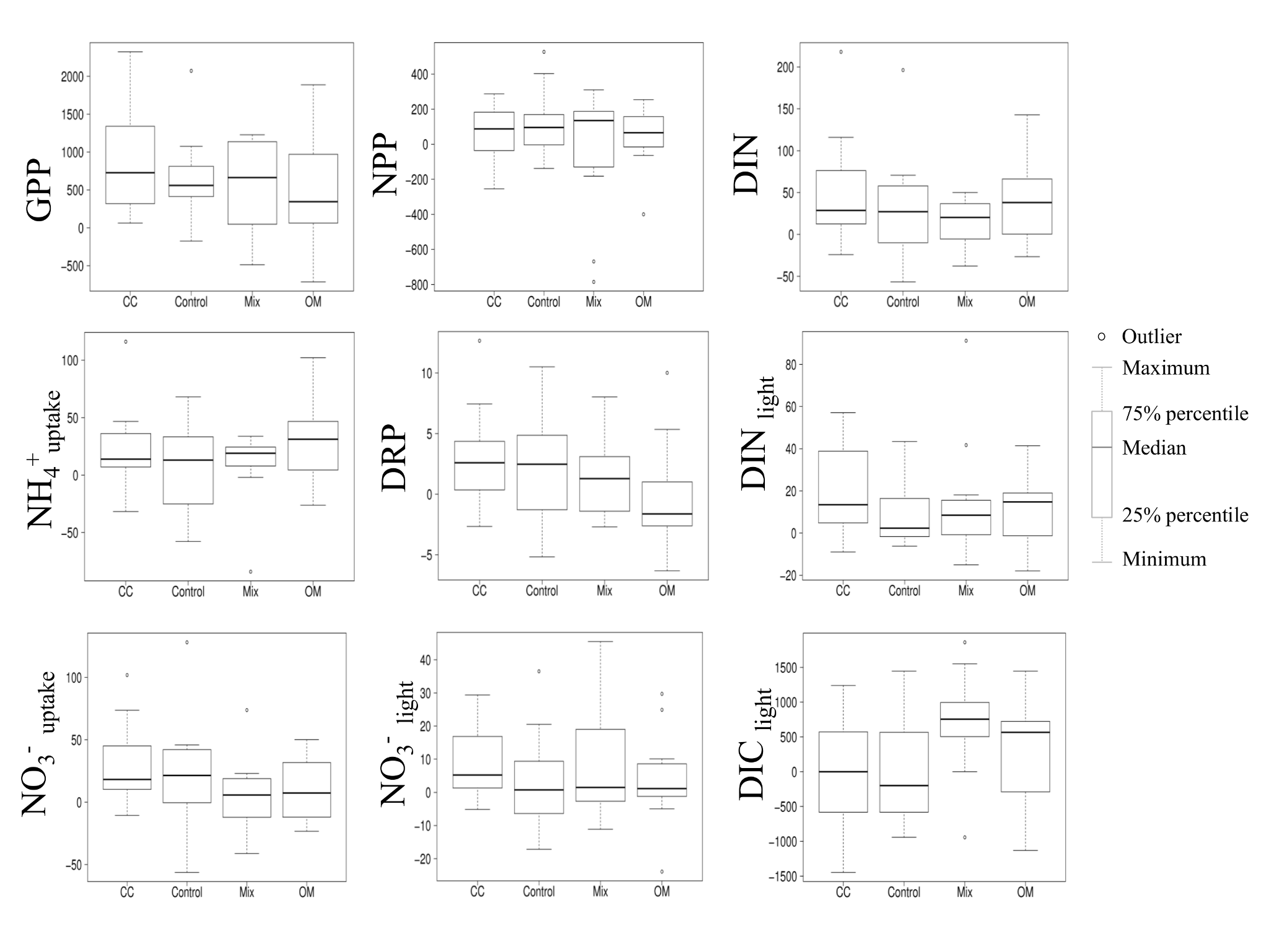

Supplement: Figure S2 — Boxplots showing the variability of the oxygen and nutrient fluxes through the four treatments (Calcium carbonate, Control, Mix and Organic matter). Gross primary production (GPP), net primary production (NPP), ammonium uptake (NH4 +), dissolved reactive phosphorus (DRP), dissolved inorganic nitrogen uptake (DINuptake), dissolved inorganic nitrogen in daylight (DINlight), nitrate uptake and during daylight (NO3 − uptake and NO3 − light), dissolved inorganic carbon during daylight (DIClight). Units: µmol m−2 h−1. (TIF) [file pone.0081646.s002.tif]

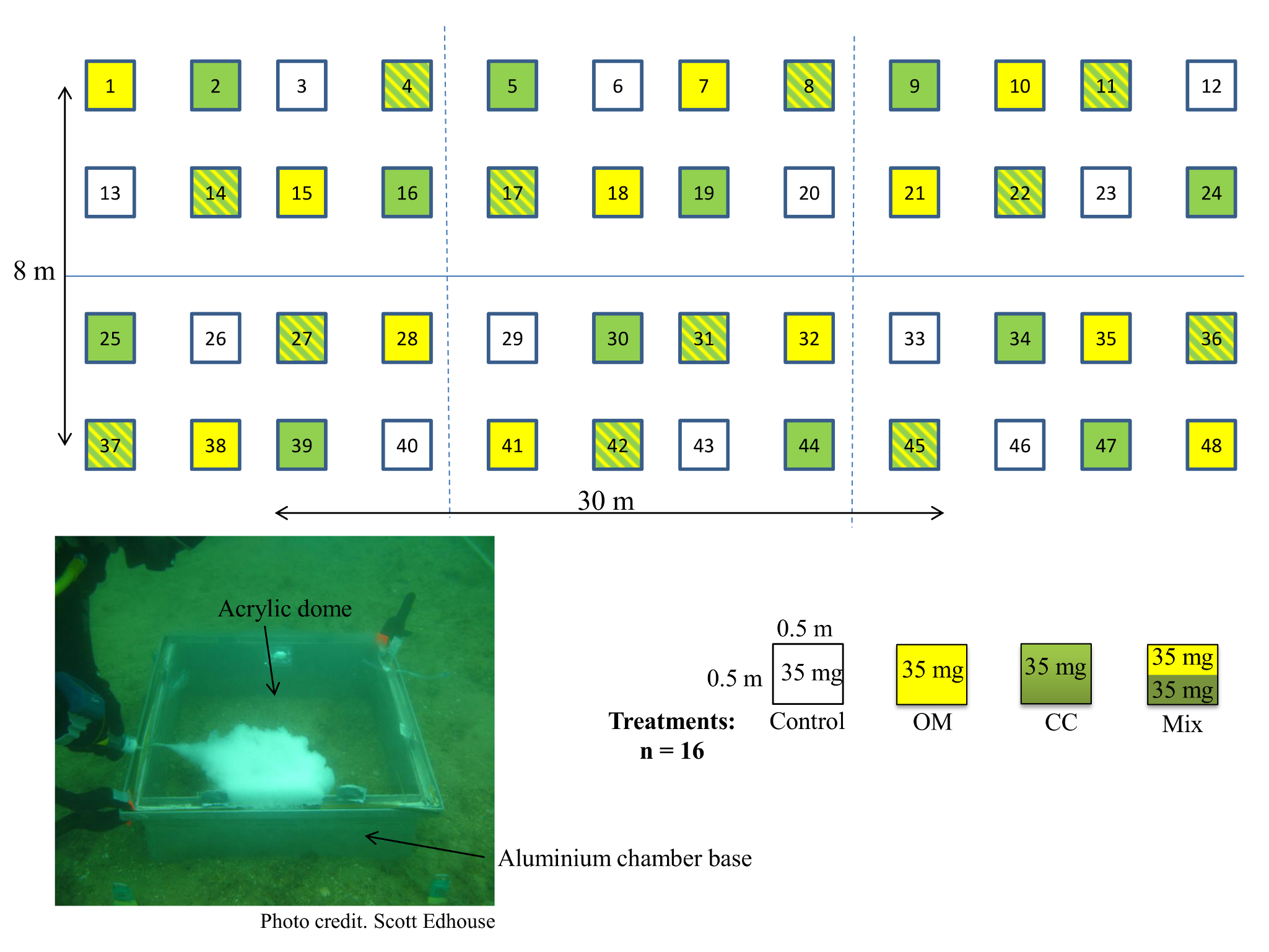

Supplement: Figure S3 — Schematic diagram showing the layout of the plots with the treatments (Calcium carbonate, Organic matter, Mix: OM+CC, and Control) across the sampling area in a balanced orthogonal design with randomized interspersed treatment positions (3 blocks). Below a picture showing a diver introducing the treatment inside one of the plots through a syringe activated sampling port. Plots were made of two parts: a square chamber base (aluminum border with sides 50×50 cm×10 cm tall) pressed down into the sediment (∼5 cm) and a clear acrylic plastic dome (50×50 cm) fitted to the chamber bases and clamped in place. (TIF) [file pone.0081646.s003.tif]
