# Supplementary material for: Sensitivity of Heterogeneous Marine Benthic Habitats to Subtle Stressors
Source: PLoS One. 2013 Nov 28;8(11):e81646. doi: 10.1371/journal.pone.0081646 (PMC3842950; doi:10.1371/journal.pone.0081646)
Supplement: Table S1 — Summary of the ANOVAs (Treatment and blocks as fixed factors) for sediment characteristics (chlorophyll a and organic matter), water pH and macrofauna communities (Abundance of large organisms, total abundance, number of species and the abundance of deposit and suspension feeders). Treatments: OM: Organic matter, CC: Calcium carbonate, Mix: OM+CC, Control. Significant results in italics. (DOCX) [file pone.0081646.s004.docx]

**Table S1.** Summary of the ANOVAs (Treatment and blocks as fixed factors) for sediment characteristics (chlorophyll a and organic matter), water pH and macrofauna communities (Abundance of large organisms, total abundance, number of species and the abundance of deposit and suspension feeders) over establishment (Block 1) and sampling date (Block 2). Treatments: OM: Organic matter, CC: Calcium carbonate, Mix: OM + CC, Control. Significant results in italics.

|  |  | Chla |  | | OM |  | pH |  |  |  |  |  |
| --- | --- | --- | --- | --- | --- | --- | --- | --- | --- | --- | --- | --- |
| Source | df | MS | F | | MS | F | MS | F |  |  |  |  |
| Treatment | 3 | 1.48 | 1.64 | | 0.1 | 0.68 | 0.0002 | 0.2 |  |  |  |  |
| Block1^a^ | 1 | 2.99 | 3.32 | | 1.03 | *9.55^**^* | 0.01 | *10.3^**^* |  |  |  |  |
| Treatment*Block1 | 2 | 0.65 | 0.72 | | 0.03 | 0.28 | 0.0001 | 0.1 |  |  |  |  |
| Residuals | 40 | 0.9 |  | | 0.11 |  | 0.0008 |  |  |  |  |  |
| Treatment | 3 | 1.48 | 1.7 | | 0.07 | 0.7 | 0.0002 | 0.42 |  |  |  |  |
| Block2^b^ | 2 | 1.07 | 1.23 | | 0.81 | *7.66^**^* | 0.01 | *35.3^***^* |  |  |  |  |
| Treatment*Block2 | 6 | 1.25 | 1.43 | | 0.003 | 0.03 | 0.0001 | 1.15 |  |  |  |  |
| Residuals | 36 | 0.87 |  | | 0.1 |  | 0.0004 |  |  |  |  |  |
|  |  | Abundance large | | | Total Abundance | | Species richness | | Deposit feeders | | Suspension feeders | |
| Source | df | MS | | F | MS | F | MS | F | MS | F | MS | F |
| Treatment | 3 | 10.5 | | 1.02 | 818.3 | 1.71 | 29.9 | 1.4 | 125.9 | 2.71 | 129.4 | 0.71 |
| Block1^a^ | 1 | 22.7 | | 2.21 | 385.3 | 0.8 | 1.3 | 0.1 | 63 | 1.36 | 80.1 | 0.44 |
| Treatment*Block1 | 2 | 7.85 | | 0.76 | 380.7 | 0.79 | 33.6 | 1.6 | 78.7 | 1.7 | 452.9 | 2.49 |
| Residuals | 40 | 10.3 | |  | 479.5 |  | 21.4 |  | 46.4 |  | 182 |  |
| Treatment | 3 | 10.5 | | 0.92 | 818.3 | 1.75 | 29.9 | 1.46 | 125.9 | 2.81 | 129.4 | 0.62 |
| Block2^b^ | 2 | 7.02 | | 0.61 | 718.1 | 1.53 | 21 | 1.03 | 93.8 | 2.1 | 173.5 | 0.83 |
| Treatment*Block2 | 6 | 5.2 | | 0.84 | 401.9 | 0.86 | 29.8 | 1.45 | 58.9 | 1.31 | 142.9 | 0.68 |
| Residuals | 36 | 11.5 | |  | 468.4 |  | 20.5 |  | 44.9 |  | 208.8 |  |
| ^a^ Block1 = Establishment date (2 levels: eastern *vs* western halves of the sampling array, see Figure S3 for a layout of the plots) | | | | | | | | | | | | |
| ^b^ Block2 = Sampling date (3 levels: eastern, middle and western thirds of the sampling array, see Figure S3)  Significant results: ^+^ 0.10 < p < 0.05; *p < 0.05; **p < 0.01; ***p < 0.001 | | | | | | | | | | | | |
