# Supplementary material for: Sensitivity of Heterogeneous Marine Benthic Habitats to Subtle Stressors
Source: PLoS One. 2013 Nov 28;8(11):e81646. doi: 10.1371/journal.pone.0081646 (PMC3842950; doi:10.1371/journal.pone.0081646)
Supplement: Table S2 — Summary of the ANOVA outputs from the Generalized Linear Models ( Table 1 ), including Treatment and Day (3 sampling days) as fixed factors. (DOCX) [file pone.0081646.s005.docx]

**Table S2.** Summary of the ANOVA outputs from all the Generalized Linear Models (Table 1), including Treatment and Day (3 sampling days) as fixed factors.

| Model summary: GPP ~NH_4_^+^*Treatment*Day | | | |  |  |
| --- | --- | --- | --- | --- | --- |
| Null | df | Deviance | Rsdf | RsDev | F |
| NH_4_^+^ | 1 | 9601536 | 43 | 9960327 | *63.6^***^* |
| Treatment | 3 | 2009208 | 40 | 7951120 | *4.44^*^* |
| Day | 1 | 637083 | 39 | 7314037 | 4.22 |
| NH_4_^+^*Treatment | 3 | 1446388 | 36 | 5867649 | *3.2** |
| NH_4_^+^*Day | 1 | 1083678 | 35 | 4783971 | *7.18** |
| Treatment*Day | 3 | 29099 | 32 | 4754873 | 0.06 |
| NH_4_^+^*Treatment*Day | 3 | 377386 | 29 | 4377487 | 0.83 |
| Model summary: DIN ~DRP*Treatment*Day | | | |  |  |
| DRP | 1 | 28797.2 | 46 | 109815 | *19.9^***^* |
| Treatment | 3 | 7294.1 | 43 | 102521 | 1.7 |
| Day | 1 | 16258.9 | 42 | 86262 | *11.3^**^* |
| DRP*Treatment | 3 | 22878.8 | 39 | 63383 | *5.3^**^* |
| DRP*Day | 1 | 3840.6 | 38 | 59543 | 2.7 |
| Treatment*Day | 3 | 2424.2 | 35 | 57119 | 0.6 |
| DRP*Treatment*Day | 3 | 10997.9 | 32 | 46121 | 2.54 |
| Model summary: Chla ~Abundance*Treatment*Day | | | | |  |
| Abundance | 1 | 4.04 | 46 | 41.4 | *6.4^*^* |
| Treatment | 3 | 2.65 | 43 | 38.7 | 1.4 |
| Day | 1 | 3.3 | 42 | 35.5 | *5.2^*^* |
| Abundance*Treatment | 3 | 9.4 | 39 | 26.1 | *4.9^**^* |
| Abundance*Day | 1 | 0.11 | 38 | 25.9 | 0.2 |
| Treatment*Day | 3 | 1.9 | 35 | 24.1 | 0.99 |
| Abundance*Treatment*Day | 3 | 3.8 | 32 | 20.3 | 1.9 |
| Model summary: Chla~DF*Treatment*Day | | |  |  |  |
| DF | 1 | 0.15 | 45 | 42.9 | 0.2 |
| Treatment | 3 | 3.85 | 42 | 39.1 | 1.8 |
| Day | 1 | 3.1 | 41 | 35.9 | *4.3^*^* |
| DF*Treatment | 3 | 11.1 | 38 | 24.8 | *5.1^**^* |
| DF*Day | 1 | 0.004 | 37 | 24.8 | 0.01 |
| Treatment*Day | 3 | 0.7 | 34 | 24.1 | 0.33 |
| DF*Treatment*Day | 3 | 1.5 | 31 | 22.6 | 0.7 |
| Model summary: NO_3_^-^light ~DF*Treatment*Day | | | |  |  |
| DF | 1 | 874.1 | 45 | 9271.6 | *5.9^*^* |
| Treatment | 3 | 555.5 | 42 | 8716.1 | 1.3 |
| Day | 1 | 248.2 | 41 | 8468 | 1.7 |
| DF*Treatment | 3 | 1780.4 | 38 | 6687.6 | *4.04^*^* |
| DF*Day | 1 | 636.1 | 37 | 6051.5 | 4.3^+^ |
| Treatment*Day | 3 | 675.1 | 34 | 5376.4 | 1.53 |
| DF*Treatment*Day | 3 | 825.7 | 31 | 4550.7 | 1.9 |

Significant results in italics: ^+^ 0.10 < p < 0.05; *p < 0.05; **p < 0.01; ***p < 0.001.

GPP: gross primary production; NH_4_^+^: ammonium uptake; DIN: dissolved inorganic nitrogen; DRP: dissolved reactive phosphorus; NO_3_^-^_light_: nitrate flux during daylight; Chla: chlorophyll *a* concentration; Abundance: total macrofauna abundance; DF: deposit feeders abundance.
