# Supplementary material for: Sensitivity of Heterogeneous Marine Benthic Habitats to Subtle Stressors
Source: PLoS One. 2013 Nov 28;8(11):e81646. doi: 10.1371/journal.pone.0081646 (PMC3842950; doi:10.1371/journal.pone.0081646)
Supplement: Table S3 — Generalized Linear Model summary (regression-based models with Gaussian distribution and identity link function) indicating the significance of ammonium uptake (NH4+) on gross primary production (GPP) among treatments (OM: Organic matter, CC: Calcium carbonate, Mix: OM+CC, Control). (DOCX) [file pone.0081646.s006.docx]

**Table S3.** Generalized Linear Model summary (regression-based models with Gaussian distribution and identity link function) indicating the significance of ammonium uptake (NH_4_^+^) on gross primary production (GPP) among treatments (OM: Organic matter, CC: Calcium carbonate, Mix: OM + CC, Control).

| Model summary: GPP ~NH_4_^+^ + Treatment | | | | |
| --- | --- | --- | --- | --- |
| Coefficients | Estimate | Standard Error | t | p |
| Intercept (Control) | 602.5 | 141.5 | 4.3 | *<0.001^***^* |
| NH_4_^+^ | 15.8 | 2.2 | 7.3 | *<0.001^***^* |
| Tr(OM) | -507.6 | 197.6 | -2.6 | *0.014^*^* |
| Tr(CC) | -60.9 | 194.1 | -0.3 | 0.755 |
| Tr(Mix) | -395.6 | 196.9 | -2.0 | *0.05^+^* |
| Intercept (OM) | 94.9 | 143.1 | 0.66 | 0.511 |
| NH_4_^+^ | 15.8 | 2.2 | 7.3 | *<0.001^***^* |
| Tr(Control) | 507.6 | 197.5 | 2.6 | *0.014^*^* |
| Tr(CC) | 446.6 | 182.7 | 2.5 | *0.019^*^* |
| Tr(Mix) | 112.0 | 187.4 | 0.6 | 0.553 |
| Intercept (CC) | 541.5 | 136.9 | 3.9 | *<0.001^***^* |
| NH_4_^+^ | 15.8 | 2.2 | 7.3 | *<0.001^***^* |
| Tr(Control) | -446.6 | 182.7 | -2.5 | 0.755 |
| Tr(OM) | 60.9 | 194.1 | 0.3 | 0.019^*^ |
| Tr(Mix) | -334.6 | 186.2 | -1.8 | 0.07*^+^* |

Significant results in italics: ^+^ 0.10 < p < 0.05; *p < 0.05; **p < 0.01; ***p < 0.001.
