# Supplementary material for: Sensitivity of Heterogeneous Marine Benthic Habitats to Subtle Stressors
Source: PLoS One. 2013 Nov 28;8(11):e81646. doi: 10.1371/journal.pone.0081646 (PMC3842950; doi:10.1371/journal.pone.0081646)
Supplement: Table S4 — Generalized Linear Model summary (regression-based models with Gaussian distribution and identity link function) indicating the significance of dissolved reactive phosphorus (DRP) on dissolved inorganic nitrogen (DIN) among treatments (OM: Organic matter, CC: Calcium carbonate, Mix: OM+CC, Control). (DOCX) [file pone.0081646.s007.docx]

**Table S4.** Generalized Linear Model summary (regression-based models with Gaussian distribution and identity link function) indicating the significance of dissolved reactive phosphorus (DRP) on dissolved inorganic nitrogen (DIN) among treatments (OM: Organic matter, CC: Calcium carbonate, Mix: OM + CC, Control).

| Model summary: DIN ~DRP*Treatment | | | | |
| --- | --- | --- | --- | --- |
| Coefficients | Estimate | Standard Error | t | p |
| Intercept (Control) | 3.81 | 15.1 | 0.25 | 0.802 |
| DRP | 11.4 | 2.9 | 3.92 | *<0.001^***^* |
| Tr(OM) | 34.5 | 19.5 | 1.77 | *0.086^+^* |
| Tr(CC) | 10.1 | 21.4 | 0.47 | 0.639 |
| Tr(Mix) | 17.4 | 21.0 | 0.83 | 0.413 |
| DRP*Tr(OM) | -13.8 | 4.2 | -3.31 | *0.002^**^* |
| DRP*Tr(CC) | 0.94 | 4.2 | 0.22 | 0.824 |
| DRP*Tr(Mix) | -7.15 | 6.7 | -1.1 | 0.291 |
| Intercept (OM) | 38.31 | 12.4 | 3.10 | *0.004^**^* |
| DRP | -2.4 | 3.0 | -0.81 | 0.426 |
| Tr(Control) | -34.5 | 19.5 | -1.77 | 0.085 |
| Tr(CC) | -24.4 | 19.7 | -1.24 | 0.224 |
| Tr(Mix) | -17.1 | 19.2 | -0.89 | 0.378 |
| DRP*Tr(Control) | 13.8 | 4.2 | 3.31 | *0.002^**^* |
| DRP*Tr(CC) | 14.80 | 4.3 | 3.45 | *0.001^**^* |
| DRP*Tr(Mix) | 6.66 | 6.7 | 0.99 | 0.328 |
| Intercept (CC) | 13.94 | 15.3 | 0.91 | 0.367 |
| DRP | 12.4 | 3.1 | 4.03 | *0.0003^***^* |
| Tr(Control) | -10.1 | 21.4 | -0.47 | 0.639 |
| Tr(OM) | 24.4 | 19.7 | 1.24 | 0.224 |
| Tr(Mix) | 7.2 | 21.1 | 0.34 | 0.734 |
| DRP*Tr(Control) | -0.9 | 4.2 | -0.22 | 0.824 |
| DRP*Tr(OM) | -14.80 | 4.2 | -3.45 | *0.002^**^* |
| DRP*Tr(Mix) | -8.10 | 6.8 | -1.2 | 0.238 |

Significant results in italics: ^+^ 0.10 < p < 0.05; *p < 0.05; **p < 0.01; ***p < 0.001.
