# Supplementary material for: Sensitivity of Heterogeneous Marine Benthic Habitats to Subtle Stressors
Source: PLoS One. 2013 Nov 28;8(11):e81646. doi: 10.1371/journal.pone.0081646 (PMC3842950; doi:10.1371/journal.pone.0081646)
Supplement: Table S5 — Generalized Linear Model summary (regression-based models with Gaussian distribution and identity link function) indicating the significance of chlorophyll a content on the total macrofauna abundance and the abundance of deposit feeders among treatments (OM: Organic matter, CC: Calcium carbonate, Mix: OM+CC, Control). SE: Standard Error. (DOCX) [file pone.0081646.s008.docx]

**Table S5.** Generalized Linear Model summaries (regression-based models with Gaussian distribution and identity link function) indicating the significance of chlorophyll *a* content on the total macrofauna abundance and the abundance of deposit feeders among treatments (OM: Organic matter, CC: Calcium carbonate, Mix: OM + CC, Control). SE: Standard Error.

| Model summary: Chla~Abundance*Treatment | | | | |  | Model summary: Chla~DF*Treatment | | | | |
| --- | --- | --- | --- | --- | --- | --- | --- | --- | --- | --- |
| Coefficients | Estimate | SE | t | p |  | Coefficients | Estimate | SE | t | p |
| Intercept (Control) | 2.1 | 1.13 | 1.84 | *0.07^+^* |  | Intercept (Control) | 1.97 | 0.5 | 3.62 | *<0.001**** |
| Abundance | 0.02 | 0.02 | 0.3 | 0.299 |  | DF | 0.13 | 0.1 | 2.59 | *0.01*** |
| Tr(OM) | -0.3 | 1.56 | -0.19 | 0.847 |  | Tr(OM) | 0.27 | 0.8 | 0.34 | 0.734 |
| Tr(CC) | 3.28 | 1.37 | 2.4 | *0.022^*^* |  | Tr(CC) | 2.27 | 0.8 | 3.02 | *0.004*** |
| Tr(Mix) | 1.79 | 1.49 | 1.2 | 0.236 |  | Tr(Mix) | 0.54 | 0.8 | 0.68 | 0.503 |
| Abund*Tr(OM) | -0.01 | 0.02 | -0.44 | 0.663 |  | DF*Tr(OM) | -0.11 | 0.1 | -1.89 | *0.066^+^* |
| Abund*Tr(CC) | -0.05 | 0.02 | -2.43 | *0.02^*^* |  | DF*Tr(CC) | -0.2 | 0.1 | -3.49 | *0.001^**^* |
| Abund*Tr(Mix) | -0.03 | 0.021 | -1.6 | 0.120 |  | DF*Tr(Mix) | -0.1 | 0.1 | -1.39 | 0.172 |
| Intercept (OM) | 1.77 | 1.08 | 1.64 | 0.110 |  | Intercept (OM) | 2.24 | 0.6 | 3.86 | *0.0004^***^* |
| Abundance | 0.01 | 0.012 | 0.697 | 0.490 |  | DF | 0.015 | 0 | 0.48 | 0.631 |
| Tr(Control) | 0.3 | 0.304 | 0.195 | 0.847 |  | Tr(Control) | -0.27 | 0.8 | -0.34 | 0.734 |
| Tr(CC) | 3.586 | 1.34 | 2.68 | *0.011^*^* |  | Tr(CC) | 1.99 | 0.8 | 2.57 | *0.014^*^* |
| Tr(Mix) | 2.091 | 1.45 | 1.44 | 0.157 |  | Tr(Mix) | 0.27 | 0.8 | 0.325 | 0.747 |
| Abund*Tr(Control) | 0.01 | 0.021 | 0.44 | 0.663 |  | DF*Tr(Control) | 0.111 | 0.1 | 1.89 | *0.066^+^* |
| Abund*Tr(CC) | -0.04 | 0.016 | -2.42 | *0.02^*^* |  | DF*Tr(CC) | -0.09 | 0 | -2.1 | *0.047^*^* |
| Abund*Tr(Mix) | -0.02 | 0.018 | -1.38 | 0.176 |  | DF*Tr(Mix) | 0.013 | 0.1 | 0.22 | 0.827 |
| Intercept (CC) | 5.36 | 0.788 | 6.79 | *3.6e-08^**^* |  | Intercept (CC) | 4.24 | 0.5 | 8.23 | *4.7e-10^***^* |
| Abundance | -0.03 | 0.01 | -2.96 | *0.005^**^* |  | DF | -0.078 | 0 | -2.42 | *0.02^*^* |
| Tr(Control) | -3.3 | 1.37 | -2.39 | *0.02^*^* |  | Tr(Control) | -2.27 | 0.8 | -3.02 | *0.004^**^* |
| Tr(OM) | -3.59 | 1.34 | -2.68 | *0.011^*^* |  | Tr(OM) | -1.99 | 0.8 | -2.57 | *0.014^*^* |
| Tr(Mix) | -1.5 | 1.25 | -1.2 | 0.238 |  | Tr(Mix) | -1.725 | 0.8 | -2.21 | *0.033^*^* |
| Abund*Tr(Control) | 0.05 | 0.02 | 2.43 | *0.02^*^* |  | DF*Tr(Control) | 0.204 | 0.1 | 3.5 | *0.0012^**^* |
| Abund*Tr(OM) | 0.039 | 0.015 | 2.42 | *0.02^*^* |  | DF*Tr(OM) | 0.093 | 0.1 | 2.1 | *0.04^*^* |
| Abund*Tr(Mix) | 0.014 | 0.016 | 0.89 | 0.381 |  | DF*Tr(Mix) | 0.106 | 0.1 | 1.79 | 0.082 |

Significant results in italics: ^+^ 0.10 < p < 0.05; *p < 0.05; **p < 0.01; ***p < 0.001.
