# Supplementary material for: Sensitivity of Heterogeneous Marine Benthic Habitats to Subtle Stressors
Source: PLoS One. 2013 Nov 28;8(11):e81646. doi: 10.1371/journal.pone.0081646 (PMC3842950; doi:10.1371/journal.pone.0081646)
Supplement: Table S6 — Generalized Linear Model summary (regression-based models with Gaussian distribution and identity link function) indicating the significance of NO3− flux during daylight on the abundance of deposit feeders (DF) among treatments (OM: Organic matter, CC: Calcium carbonate, Mix: OM+CC, Control). (DOCX) [file pone.0081646.s009.docx]

**Table S6.** Generalized Linear Model summary (regression-based models with Gaussian distribution and identity link function) indicating the significance of NO_3_^-^ flux during daylight on the abundance of deposit feeders (DF) among treatments (OM: Organic matter, CC: Calcium carbonate, Mix: OM + CC, Control).

| Model summary: NO_3_^-^_light_~DF*Treatment | | | | |
| --- | --- | --- | --- | --- |
| Coefficients | Estimate | Standard Error | t | *p* |
| Intercept (Control) | 12.93 | 8.63 | 1.5 | 0.142 |
| DF | -1.01 | 0.77 | -1.31 | 0.199 |
| Tr(OM) | 9.5 | 12.6 | 0.75 | 0.457 |
| Tr(CC) | -8.83 | 11.9 | -0.74 | 0.461 |
| Tr(Mix) | 15.7 | 12.7 | 1.24 | 0.222 |
| DF^*^Tr(OM) | -0.14 | 0.93 | -0.15 | 0.882 |
| DF*Tr(CC) | 1.4 | 0.93 | 1.5 | 0.142 |
| DF*Tr(Mix) | -0.8 | 1.11 | -0.71 | 0.483 |
| Intercept (OM) | 22.4 | 9.22 | 2.43 | *0.02^*^* |
| DF | -1.2 | 0.51 | -2.25 | 0.03* |
| Tr(Control) | -9.5 | 12.6 | -0.75 | 0.457 |
| Tr(CC) | -18.33 | 12.3 | -1.49 | 0.144 |
| Tr(Mix) | 6.24 | 13.1 | 0.48 | 0.636 |
| DF^*^Tr(Control) | 0.14 | 0.93 | 0.15 | 0.882 |
| DF*Tr(CC) | 1.53 | 0.72 | 2.11 | *0.041^*^* |
| DF*Tr(Mix) | -0.65 | 0.95 | -0.68 | 0.499 |
| Intercept (CC) | 4.1 | 8.15 | 0.5 | 0.619 |
| DF | 0.38 | 0.51 | 0.74 | 0.465 |
| Tr(Control) | 8.8 | 11.9 | 0.74 | 0.461 |
| Tr(OM) | 18.3 | 12.3 | 1.5 | 0.144 |
| Tr(Mix) | 24.6 | 12.4 | 1.99 | *0.05^+^* |
| DF^*^Tr(Control) | -1.4 | 0.93 | -1.5 | 0.142 |
| DF*Tr(OM) | -1.53 | 0.72 | -2.11 | *0.041^*^* |
| DF*Tr(Mix) | -2.2 | 0.95 | -2.3 | *0.027^*^* |

Significant results in italics: ^+^ 0.10 < p < 0.05; *p < 0.05; **p < 0.01; ***p < 0.001.
